# Supplementary material for: Hot spot prediction in protein-protein interactions by an ensemble system
Source: BMC Syst Biol. 2018 Dec 31;12(Suppl 9):132. doi: 10.1186/s12918-018-0665-8 (PMC6311905; doi:10.1186/s12918-018-0665-8)
Supplement: Supplementary file 2 — All relevant MATLAB code. A simple Matlab implement of our predictor. (ZIP 19,742 kb) [file 12918_2018_665_MOESM2_ESM.zip › Additional file 2/Read ASA/ASA Property/SKEMPI/1AK4D.txt]

 NetSurfP - Protein Surface Accessibility and Secondary Structure Predictions

  

|  |  |  |
| --- | --- | --- |
|  |  | NetSurfP - Protein Surface Accessibility and Secondary Structure Predictions  Technical University of Denmark |

  


---

```
# For publication of results, please cite:
# A generic method for assignment of reliability scores applied to solvent accessibility predictions.
# Bent Petersen, Thomas Nordahl Petersen, Pernille Andersen, Morten Nielsen and Claus Lundegaard
# BMC Structural Biology 2009, 9:51 doi:10.1186/1472-6807-9-51
#
# Column 1: Class assignment - B for buried or E for Exposed - Threshold: 25% exposure, but not based on RSA
# Column 2: Amino acid
# Column 3: Sequence name
# Column 4: Amino acid number
# Column 5: Relative Surface Accessibility - RSA
# Column 6: Absolute Surface Accessibility
# Column 7: Z-fit score for RSA prediction
# Column 8: Probability for Alpha-Helix
# Column 9: Probability for Beta-strand
# Column 10: Probability for Coil
E P  1AK4D                  1    0.569  80.684  -0.522   0.003   0.003   0.994
E I  1AK4D                  2    0.315  58.275  -0.735   0.004   0.616   0.381
B V  1AK4D                  3    0.237  36.350  -0.613   0.001   0.900   0.099
E Q  1AK4D                  4    0.346  61.778   0.169   0.001   0.900   0.099
E N  1AK4D                  5    0.326  47.726  -0.432   0.003   0.718   0.279
B L  1AK4D                  6    0.290  53.026  -0.622   0.005   0.262   0.733
E Q  1AK4D                  7    0.529  94.426  -1.516   0.005   0.045   0.951
E G  1AK4D                  8    0.424  33.377  -1.518   0.018   0.088   0.893
E Q  1AK4D                  9    0.354  63.153  -0.363   0.021   0.451   0.528
B M  1AK4D                 10    0.296  59.230   0.277   0.021   0.756   0.223
B V  1AK4D                 11    0.262  40.239   0.013   0.018   0.846   0.136
B H  1AK4D                 12    0.286  51.987  -0.068   0.018   0.846   0.136
E Q  1AK4D                 13    0.300  53.526   0.867   0.004   0.616   0.381
E A  1AK4D                 14    0.307  33.875  -1.050   0.004   0.514   0.481
B I  1AK4D                 15    0.072  13.246  -0.022   0.019   0.141   0.840
E S  1AK4D                 16    0.272  31.878   0.547   0.018   0.047   0.935
B P  1AK4D                 17    0.190  26.890  -0.630   0.782   0.003   0.216
E R  1AK4D                 18    0.363  83.035  -0.587   0.858   0.002   0.139
B T  1AK4D                 19    0.113  15.631  -0.047   0.858   0.002   0.139
B L  1AK4D                 20    0.042   7.709   0.582   0.970   0.001   0.030
E N  1AK4D                 21    0.302  44.154   0.681   0.970   0.001   0.030
B A  1AK4D                 22    0.161  17.786   1.047   0.988   0.000   0.012
B W  1AK4D                 23    0.031   7.504   0.205   0.988   0.000   0.012
B V  1AK4D                 24    0.059   9.130   0.932   0.988   0.000   0.012
E K  1AK4D                 25    0.416  85.489   1.909   0.988   0.000   0.012
B V  1AK4D                 26    0.108  16.646   1.553   0.970   0.001   0.030
B V  1AK4D                 27    0.033   5.072   0.434   0.975   0.003   0.022
E E  1AK4D                 28    0.415  72.501   1.090   0.938   0.007   0.055
E E  1AK4D                 29    0.586 102.409   0.074   0.694   0.003   0.303
E K  1AK4D                 30    0.543 111.777  -0.084   0.246   0.004   0.750
E A  1AK4D                 31    0.462  50.890  -0.762   0.053   0.005   0.942
B F  1AK4D                 32    0.218  43.733  -1.013   0.053   0.005   0.942
E S  1AK4D                 33    0.415  48.638  -0.001   0.109   0.005   0.886
E P  1AK4D                 34    0.419  59.442  -0.501   0.622   0.015   0.363
E E  1AK4D                 35    0.289  50.541  -0.039   0.660   0.049   0.291
B V  1AK4D                 36    0.064   9.868  -0.136   0.701   0.107   0.192
B I  1AK4D                 37    0.123  22.663   1.137   0.701   0.107   0.192
B P  1AK4D                 38    0.214  30.409   0.377   0.831   0.044   0.125
B M  1AK4D                 39    0.087  17.509   0.033   0.879   0.010   0.111
B F  1AK4D                 40    0.044   8.771   0.125   0.879   0.010   0.111
B S  1AK4D                 41    0.135  15.845   0.100   0.879   0.010   0.111
B A  1AK4D                 42    0.085   9.400   0.116   0.879   0.010   0.111
B L  1AK4D                 43    0.086  15.728   0.215   0.879   0.010   0.111
B S  1AK4D                 44    0.095  11.122   0.194   0.622   0.015   0.363
E E  1AK4D                 45    0.447  78.178  -0.107   0.257   0.016   0.727
E G  1AK4D                 46    0.450  35.384  -0.839   0.058   0.017   0.925
B A  1AK4D                 47    0.062   6.832  -0.493   0.018   0.019   0.964
B T  1AK4D                 48    0.202  27.976   0.926   0.058   0.017   0.925
B P  1AK4D                 49    0.130  18.419  -0.761   0.522   0.016   0.462
E Q  1AK4D                 50    0.351  62.724   0.718   0.717   0.014   0.269
B D  1AK4D                 51    0.169  24.338  -1.066   0.622   0.015   0.363
B L  1AK4D                 52    0.039   7.141  -0.008   0.923   0.002   0.076
B N  1AK4D                 53    0.222  32.501   0.341   0.970   0.001   0.030
B T  1AK4D                 54    0.234  32.428   0.466   0.970   0.001   0.030
B M  1AK4D                 55    0.038   7.644  -0.151   0.970   0.001   0.030
B L  1AK4D                 56    0.041   7.507  -0.028   0.923   0.002   0.076
E N  1AK4D                 57    0.301  44.022   0.094   0.858   0.002   0.139
B T  1AK4D                 58    0.077  10.708  -0.254   0.694   0.003   0.303
B V  1AK4D                 59    0.083  12.834   0.240   0.430   0.016   0.555
B G  1AK4D                 60    0.256  20.171  -1.642   0.176   0.004   0.820
E G  1AK4D                 61    0.403  31.700  -1.204   0.109   0.005   0.886
B H  1AK4D                 62    0.168  30.505  -0.252   0.246   0.004   0.750
E Q  1AK4D                 63    0.462  82.567   0.132   0.694   0.003   0.303
B A  1AK4D                 64    0.273  30.118  -1.122   0.694   0.003   0.303
B A  1AK4D                 65    0.066   7.317  -0.805   0.858   0.002   0.139
B M  1AK4D                 66    0.178  35.658   0.637   0.923   0.002   0.076
E Q  1AK4D                 67    0.427  76.298   1.119   0.970   0.001   0.030
B M  1AK4D                 68    0.173  34.677   0.333   0.970   0.001   0.030
B L  1AK4D                 69    0.022   3.937   0.951   0.970   0.001   0.030
E K  1AK4D                 70    0.344  70.740   1.249   0.970   0.001   0.030
E E  1AK4D                 71    0.537  93.884   0.122   0.923   0.002   0.076
B T  1AK4D                 72    0.211  29.238   0.985   0.923   0.002   0.076
B I  1AK4D                 73    0.057  10.527   0.852   0.858   0.002   0.139
E N  1AK4D                 74    0.437  63.962   1.451   0.858   0.002   0.139
E E  1AK4D                 75    0.605 105.763   0.855   0.923   0.002   0.076
E E  1AK4D                 76    0.352  61.494   0.536   0.923   0.002   0.076
B A  1AK4D                 77    0.138  15.153  -0.488   0.923   0.002   0.076
E A  1AK4D                 78    0.511  56.279   0.290   0.858   0.002   0.139
E E  1AK4D                 79    0.550  96.085   0.821   0.879   0.010   0.111
B W  1AK4D                 80    0.107  25.613   1.021   0.717   0.014   0.269
E D  1AK4D                 81    0.341  49.167  -0.697   0.430   0.016   0.555
E R  1AK4D                 82    0.479 109.599   0.099   0.257   0.016   0.727
E L  1AK4D                 83    0.353  64.671  -0.020   0.184   0.043   0.773
B H  1AK4D                 84    0.209  38.035   0.902   0.053   0.043   0.903
E P  1AK4D                 85    0.372  52.787  -0.643   0.018   0.047   0.935
E V  1AK4D                 86    0.363  55.855  -0.046   0.052   0.084   0.864
E H  1AK4D                 87    0.517  93.970  -1.462   0.052   0.084   0.864
E A  1AK4D                 88    0.311  34.239  -0.927   0.018   0.047   0.935
E G  1AK4D                 89    0.447  35.163  -1.202   0.018   0.019   0.964
E P  1AK4D                 90    0.401  56.959  -1.914   0.018   0.047   0.935
B I  1AK4D                 91    0.233  43.105  -1.376   0.018   0.047   0.935
E A  1AK4D                 92    0.335  36.928  -1.225   0.018   0.019   0.964
E P  1AK4D                 93    0.430  60.974  -1.793   0.018   0.019   0.964
B G  1AK4D                 94    0.299  23.508  -1.720   0.018   0.047   0.935
E Q  1AK4D                 95    0.310  55.384  -1.234   0.018   0.047   0.935
B M  1AK4D                 96    0.106  21.191  -0.747   0.018   0.047   0.935
E R  1AK4D                 97    0.455 104.081  -0.644   0.018   0.047   0.935
E E  1AK4D                 98    0.406  70.841  -0.401   0.018   0.047   0.935
B P  1AK4D                 99    0.204  28.933  -0.894   0.018   0.047   0.935
E R  1AK4D                100    0.509 116.538  -0.341   0.053   0.043   0.903
B G  1AK4D                101    0.308  24.255  -1.256   0.113   0.087   0.800
E S  1AK4D                102    0.330  38.723  -0.520   0.118   0.150   0.732
B D  1AK4D                103    0.299  43.071  -0.804   0.216   0.235   0.548
B I  1AK4D                104    0.093  17.168  -0.806   0.216   0.235   0.548
B A  1AK4D                105    0.185  20.332  -0.838   0.216   0.235   0.548
B G  1AK4D                106    0.270  21.218  -1.753   0.125   0.227   0.648
B T  1AK4D                107    0.231  32.012  -0.626   0.056   0.142   0.802
B T  1AK4D                108    0.332  46.035  -1.436   0.052   0.084   0.864
B S  1AK4D                109    0.204  23.909  -0.322   0.184   0.043   0.773
E T  1AK4D                110    0.374  51.943   0.565   0.430   0.016   0.555
B L  1AK4D                111    0.215  39.403   0.399   0.923   0.002   0.076
E Q  1AK4D                112    0.301  53.705  -0.111   0.923   0.002   0.076
E E  1AK4D                113    0.314  54.873   0.343   0.938   0.007   0.055
B Q  1AK4D                114    0.095  17.038  -0.625   0.879   0.010   0.111
B I  1AK4D                115    0.075  13.893  -0.452   0.879   0.010   0.111
E G  1AK4D                116    0.292  22.988   0.980   0.802   0.014   0.185
B W  1AK4D                117    0.129  31.049  -0.211   0.717   0.014   0.269
B M  1AK4D                118    0.122  24.412   0.093   0.622   0.015   0.363
B T  1AK4D                119    0.266  36.866  -1.338   0.339   0.016   0.645
E H  1AK4D                120    0.628 114.306  -0.986   0.109   0.005   0.886
E N  1AK4D                121    0.356  52.133  -0.912   0.016   0.005   0.979
E P  1AK4D                122    0.435  61.783  -1.322   0.018   0.019   0.964
E P  1AK4D                123    0.436  61.812  -0.852   0.053   0.043   0.903
B I  1AK4D                124    0.148  27.362   0.303   0.113   0.043   0.844
E P  1AK4D                125    0.265  37.660  -0.395   0.184   0.043   0.773
B V  1AK4D                126    0.046   7.132  -0.571   0.802   0.014   0.185
B G  1AK4D                127    0.101   7.925  -0.163   0.923   0.002   0.076
E E  1AK4D                128    0.361  63.067   0.906   0.970   0.001   0.030
B I  1AK4D                129    0.044   8.122   0.942   0.970   0.001   0.030
B Y  1AK4D                130    0.029   6.240   0.966   0.970   0.001   0.030
B K  1AK4D                131    0.160  32.953   0.724   0.970   0.001   0.030
E R  1AK4D                132    0.257  58.967   0.856   0.970   0.001   0.030
B W  1AK4D                133    0.034   8.105   0.563   0.988   0.000   0.012
B I  1AK4D                134    0.026   4.828   1.129   0.988   0.000   0.012
B I  1AK4D                135    0.049   9.121   0.321   0.988   0.000   0.012
B L  1AK4D                136    0.079  14.465   0.534   0.970   0.001   0.030
B G  1AK4D                137    0.032   2.518  -1.108   0.970   0.001   0.030
B L  1AK4D                138    0.058  10.583  -0.209   0.923   0.002   0.076
E N  1AK4D                139    0.328  48.005   0.554   0.923   0.002   0.076
E K  1AK4D                140    0.343  70.555   0.342   0.858   0.002   0.139
B I  1AK4D                141    0.062  11.544  -0.712   0.879   0.010   0.111
B V  1AK4D                142    0.238  36.534   0.039   0.751   0.050   0.199
E R  1AK4D                143    0.619 141.774   0.957   0.561   0.047   0.393
E M  1AK4D                144    0.348  69.555   0.486   0.354   0.048   0.598
E Y  1AK4D                145    0.624 133.392  -1.718   0.016   0.005   0.979

Explain the output.  Go back.
```

---
